# A qualitative study exploring high school students’ understanding of, and attitudes towards, health information and claims

**DOI:** 10.1111/hex.12562

**Published:** 2017-05-05

**Authors:** Leila Cusack, Laura N Desha, Chris B Del Mar, Tammy C Hoffmann

**Affiliations:** ^1^ Centre for Research in Evidence‐Based Practice (CREBP) Bond University Robina QLD Australia

**Keywords:** appraisal, health claims, health literacy, students

## Abstract

**Background:**

Exposure to health claims, particularly in the media and social media, is pervasive, and the information conveyed is often inaccurate, incomplete or misleading. Some young people of high school ages are already making decisions about using readily available health interventions (such as sports drinks and beauty products).Although previous research has assessed adults’ understanding of health claims, no research has examined this issue in young adults who are attending high school.

**Objective:**

To explore high school students’ understanding of, and attitudes towards, concepts relevant to assessing health information and claims.

**Design:**

A qualitative study involving semi‐structured interviews with 27 Australian high school students. Responses were recorded, transcribed and a thematic analysis performed. Three themes emerged as follows: (i) Variability in sources of health information and claims, and general understanding of their creation and accuracy of content, (ii) The use of substitute indicators to assess health information and claims and make judgements about their trustworthiness, (iii) Uncertainty about, and literal interpretation of, the language of health claims. Despite general scepticism of health claims and admitted uncertainty of research terminology, many students were generally convinced. Students had poor understanding about how health claims are generated and tended to rely on substitute indicators, such as endorsements, when evaluating the believability of claims.

**Conclusion:**

School students’ lack of awareness of basic health research processes and methods of assessing the accuracy of health information and claims makes them vulnerable to distorted and misleading health information. This restricts their ability to make informed health decisions – a skill that increases in importance as they become adults.

## 1 BACKGROUND

One consequence of the pervasive presence of mass media is that people are frequently exposed to health claims from varied sources (for example, from the Internet, television, radio and magazines). Many of these claims are inaccurate.^1^, ^2^, ^3^, ^4^, ^5^ Basing health decisions on misinformation can be harmful to a person's health and a waste of resources of both individuals and health systems. Conversely, mistrust of reliable and accurate health information can also negatively impact upon people's health and resource use.^6^, ^7^ Knowing how to assess the validity of health claims can empower people to identify accurate health information upon which to base informed decisions.

### 1.1 Assessing health claims

Health literacy encompasses the ability to gain access to, and interact with, health information in an effective manner, such that good health is promoted and maintained.^8^ Nutbeam^9^ describes health literacy abilities as ranging from basic to more advanced skills; including functional, interactive and critical health literacy.^9^

Most interventions developed to improve health literacy have focused on improving a person's functional health literacy, concentrating on skills such as basic numeracy and literacy, along with knowledge of medical conditions, ensuring the safe use of medications and effective navigation of the health‐care system.^9^ Interactive health literacy involves the combination of advanced cognitive, literacy and social skills, to enable a person to extract information from different types of communication and be adaptable in the face of new health information and circumstances.^10^ Critical health literacy comprises higher cognitive skills, enabling people to analyse health information and claims and use this information appropriately to overcome barriers to improving health and well‐being.^9^, ^11^ These specific skills enable people to assess the credibility of health information directly, rather than relying on other indicators of quality, such as the perceived authority of the authors, or source of the information.^12^, ^13^

Being able to assess claims about the effectiveness of health interventions requires underlying knowledge about the processes involved in testing health interventions and basic research concepts (such as major types of study designs; experimental vs observational studies). Furthermore, for people to be able to assess health information and claims, universally relevant key concepts^14^, ^15^, ^16^, ^17^ need to be understood, such as the need for systematic reviews; concepts such as randomization and blinding, the role of chance, placebo effects; and how to interpret results (for example, relative vs absolute risk).

### 1.2 Critical health literacy for students

As children grow older, parental involvement in decision making decreases and adolescents increasingly assume responsibility for making decisions about their health. However, adolescents may already be making decisions about broader health issues, such as consuming sports drinks, supplements, skin creams or other readily available products that claim to improve some aspect of the consumer's health.^18^ Mass media has been cited as a source for health information for adolescents, particularly those with chronic illness (eg asthma, mental illness),^19^ but without adequate preparation for interpreting information they find, students may be unable to make appropriate health decisions. While most school students have minimal interaction with the healthcare system, this will increase as they become adults. Intervening to improve critical health literacy skills, while children are at school, may foster the development of skills that are necessary for health decision making through adolescence and into adulthood.^19^

### 1.3 Education to assess health claims

Previous studies have explored adults’ awareness of some of the key concepts involved in health claim appraisal – for example, randomization,^20^, ^21^, ^22^, ^23^ double‐blinding^23^ and informed consent.^24^, ^25^, ^26^ Some studies have explored university health students’ and health professionals’ understanding of these concepts – typically as part of evidence‐based practice.^27^, ^28^, ^29^ Other studies have explored the way people assess health information and claims on the Internet; however, these studies specifically refer to assessing aspects of Internet site quality, rather than a direct assessment of the accuracy of health claims on the site.^30^, ^31^, ^32^, ^33^

Research specifically focusing on critical health literacy in young people (who are not yet adults) is less common. Previous studies have explored media literacy education,^34^ students’ understanding of the general scientific process (without health context),^35^, ^36^ and ability to assess online health information.^37^

Incorporating critical health literacy education into school curricula has the potential to widely disseminate information and expose as many students as possible to this learning opportunity.^19^ Education programmes that are designed to teach students to understand how, and why it is necessary, to assess health claims are currently being evaluated with African school students.^38^, ^39^ These programmes were developed for the eldest students at primary school (ages 10‐12 years old) as the researchers considered these students mature enough to understand the material.^40^ Beyond these studies, research into students’ understanding of aspects of critical health literacy, including interpretation of health claims, is lacking. Understanding of these aspects can be used to inform the development of school educational interventions, which aim to enable students to critically evaluate information about health interventions and make informed decisions.

### 1.4 Aims

This study aimed to explore Australian high school students’ understanding of, and attitudes towards, the concepts relevant to the assessment of health information and claims.

## 2 METHODS

### 2.1 Recruitment and participants

We focused on students in Grades 7‐9 (in Australia, these students are approximately 12‐15 years old). Purposive sampling was used to select the schools to invite for participation, with the aim of involving public (government‐funded) and private (fee‐paying) schools, and schools across a range of socioeconomic regions. Ethical approval was provided by the Human Research Ethics Committee at Bond University on the 1st of May 2015, and approval to approach public schools was granted by the relevant government education departments.

Between July and October 2015, seven Australian schools, in two Australian states, were approached to discuss potential involvement. Three agreed to participate. Once each principal had provided consent for school participation, students were recruited via internal advertising from teachers. The research was initially described to the students by the principal or nominated teacher. Students who indicated interest in participating were provided an information sheet and consent form to take home for parental completion.

### 2.2 Procedure

One author (LC) performed all of the interviews using an interview script, between August and October 2015. Each school organized a meeting room and a schedule of students. The interviewer collected the signed consent form prior to commencing, explained the interview process and expected duration (15‐20 minutes). Each interview was audio‐recorded, with participants’ consent, and later transcribed.

### 2.3 Data collection

The semi‐structured interview questions were designed to explore students’ understanding of, and assumptions regarding, the generation of information about health interventions; the role of research; how health information/claims are interpreted and/or assessed; and the perceived meaning of, and attitudes towards, associated terms (eg “evidence‐based,” “scientifically tested” and “clinically proven”). The questions were developed based upon a recently published list of key concepts that are considered important when people are assessing health claims,^14^ and a book, written with the intention of teaching and promoting critical appraisal of health interventions, particularly within the public/lay population.^15^ Throughout the interview and this study, the term “health intervention” is used broadly. This can include any intervention provided by a health professional or identified by the individual; prescription or non‐prescription; drug or non‐drug; conventional, complementary or alternative; and any product making a health claim (eg health and skin products, energy drinks, and foods). Piloting of the interview script with a convenience sample of people, who were not involved with the study, enabled subsequent refinement of the questions.

### 2.4 Data analysis

Two authors (LC, LD) independently used the process for thematic analysis outlined by Braun and Clark,^41^ whereby each familiarized themselves with the interview transcripts, and generated initial codes for overarching themes and subthemes. This process was driven by the data, and thus inductive in nature. The authors (LC, LD) iteratively compared and discussed their analyses and coding, and came to consensus on an updated coding framework, with input from another author (TH). This coding framework was independently applied by LC and LD to five randomly selected interviews before final modifications were made. After coding all of the data for interviews from three schools, LC then reviewed the coded extracts for coherency within the themes, and further refinements were made to the themes and subthemes. No further participants were recruited as data saturation was evident (no new themes emerged from analyses of the final interviews).

## 3 RESULTS

The study recruited a total of 27 students from three of the seven Australian schools approached; two in Victoria and one in Queensland. The majority of participants were girls (n=18, 67%) and in Grade 7 (16, 59%), with fewer in Grade 8 (2, 7%) or 9 (9, 33%). Private (52%) and public schools (48%) were equally represented.

### 3.1 Key themes

Analyses revealed three themes including; (i) Variability in sources of health information and claims, and general understanding of their creation and accuracy of content; (ii) The use of substitute indicators to assess health information and claims, and make judgements about their trustworthiness and (iii) Uncertainty about, and literal interpretation of, the language of health claims.

#### 3.1.1 Theme 1: Variability in sources of health information and claims, and general understanding of their creation and accuracy of content

There was great variability in students’ access to, and understanding of health information. Approximately half of the students had searched for health information on the Internet, while others indicated that they relied on their parents to provide this type of information – “If anyone in my family ever does that [searches for health information], it's my parents” (Participant 2).

Students who had searched for health information predominantly sought it from the Internet, using the search engine Google. A few sought information from medical centres or government authorities; and some from an intervention's packaging (if a physical product) – “On the back of the packet it says all the stuff that you need to look out for…” (Participant 15).

When asked where they thought people who share health information in some way (such as journalists and website writers) obtained information from, the students offered a mixed response. About half referred either to researchers, scientists, health professionals, institutions or organizations – “Through, probably, science tests and maybe they searched with professional scientists and that” (Participant 26).

Others thought this type of information came from the public, by means such as surveys, interviews or anecdotes – “Maybe from people, I guess. They might survey people” (Participant 10), or specific groups of people – “They might get them from, like, athletes or people working with athletes, like, physiotherapists and stuff like that” (Participant 7). Other students indicated that the information may have been found from another source within the mass media – “Maybe the news or the internet, the newspaper…” (Participant 16), or sought directly from manufacturers of the health products.

About half of the students made comments during the interview, which indicated basic awareness of the role of health‐related research: they were either aware of certain aspects of the research process – “[Health information comes from] scientific evidence and evidence by past experiences and about experiments, as such, and how things work and things don't work” (Participant 20), or that research has a role in generating reliable health information – “[what makes claims true is when] there is research behind [the health information] and not them just claiming” (Participant 13). Some students mentioned terms such as “evidence”, “research”, “scientists”, “experiments” or “testing”, but only a few were able to elaborate on these concepts:

Well, if they actually tested it and stuff like that, so, yeah, they actually have and they can show that they've actually tested it, and they can't just make up figures. (Participant 18)

Some of these responses appeared to have drawn on general knowledge rather than specific knowledge of the topic or health information. For example, some students mentioned that information about interventions may change over time, with one student stating that the reader would:

…never know who is writing (the information)… it could be 50 years old and a whole new discovery was made the other day. (Participant 8)

When prompted to elaborate on responses to the question, “You said that all research is probably not true, but why do you think that?”, a couple of students inadvertently referred to the influence of bias and placebo effect – “cause some people might be biased…” (Participant 24) and “…if you believe this [treatment] will help you, then it will probably help you” (Participant 15).

When asked if health information and claims were generally true, most students acknowledged that not all is:

Well, most of the time they say it's clinically proven or something, but we don't know. (Participant 2)

They can get [information] from test participants and … or general public, people who have tried it or sometimes they might even just make them up. (Participant 14)

However, although many students expressed general scepticism, some indicated they believed that health information and claims were, “basically true most of the time” (Participant 15) and generally justifiable:

…they have to get people to check it and have to go through some before they advertise it on TV to see if it's correct… like a publisher for a book or something… (Participant 11)

Some students identified a reason that health claims on the Internet may not be legitimate and can be created by people without authority or integrity:

…people can lie pretty easily. Like, it's not too hard, especially on the internet, just write a couple of words that aren't exactly true and there you go, you've got … and this amazing statement about something that is completely false… (Participant 14)

Nearly all the students identified the existence of ulterior motives or other vested interests that can be behind health information:

…different people want you to believe different things. (Participant 24)

Most of the time they're two companies or two brands competing against each other to try and get you to be convinced about what they believe and not what you believe. They're trying to pull you into what they want. (Participant 20)

More specifically, some students felt that health information was sometimes presented as a marketing technique or a form of advertising, “…so people will buy their product” (Participant 17).

When asked about the possibility of downsides of health interventions, all students acknowledged the potential for harm – “[it]… could fix something but also bring something else on, and it just doesn't tell you that necessarily” (Participant 9).

Most interview questions referred to health interventions in general, however, a few questions asked about health interventions, which described themselves “natural”. These types of interventions were generally viewed positively by most students – “[it would help], because it has natural ingredients and it's not artificial, and it would be more careful” (Participant 18). Some students perceived that natural ingredients were less likely to harm – “…because it's natural and it doesn't have all those toxins and stuff like that” (Participant 16). Others were not sure if products which claim to be “natural” could be harmful – “Maybe, like it could [be harmful] – well everyone reacts differently to stuff, but it is natural so it shouldn't be too harmful, but it might be harmful” (Participant 27).

#### 3.1.2 Theme 2: The use of substitute indicators to assess health information and claims, and make judgements about their trustworthiness

No students mentioned searching for, or using, any formal or validated methods of assessing health information or claims. Instead, students described the use of various substitute indicators to make their assessment, which included the following:

1. personal experience of the intervention:

You obviously just buy them both and see which “one”. (Participant 8)

- corroboration – that is, for specific health products, by finding multiple sources which provide information, to check or reinforce the initial information:

…if I saw something, then I would go and research it further and if other people… like, other websites are saying the same thing as, like, what every this product is saying, then I would probably believe it. (Participant 17)

- performing “research,” which students used to refer to searching on the Internet:

… research the name… something like Google… just see what articles, if there's… reviews about it and stuff like that. (Participant 14)

- evaluating the source of the information:

See if it comes from a reliable source. (Participant 7)

- the perceived quality of its presentation (eg on the Internet, or product packaging):

…if it's on just a crappy web site… or it doesn't have a brand or it's not set up properly or the information… doesn't have good grammar and just things like that that just make it not very good quality. (Participant 13)

Like, valid packaging would have, like, not, like, massive scientific words but actually give you, like, information that you can understand and not like … and dodgy would have, like, big words that are just jumbled together to make it look more scientific and more complicated than what it is. (Participant 13)

- a detailed description of how the product works:

… if [the company of the product] have a deep understanding, I tend to believe it. (Participant 11)

Because they know what they're talking about. (Participant 27)

- presentation of balanced information was important to some, particularly if potential negative effects were mentioned:

…if it says, ‘Studies show’, and maybe talks about the studies a bit and maybe also a thing which I guess could help is if it mentions some bad things about it, so the side effects, so it's not all good, good, good, because that's not all advertising, it also shows a couple of the bad things which is also, like, even though it does cause this, this and this, it is still pretty good and, yeah. (Participant 12)

- familiarity with the intervention provider or manufacturer made about half of the students feel more comfortable when making a decision about a health product:

… companies that I have heard of or have used, I know that they do work or don't work, so yeah, if I have heard of it then I might try it, but if I haven't heard of it, I still might try it, but I might have a bit of risk. (Participant 21)

Other students, however, did not use this as a substitute indicator, with some saying that they would not assume a treatment by a known brand would be better than one by an unknown brand, while others were uncertain if being familiar with a brand influenced their belief in product claims:

‘It might be, but there might be good companies that you've just never heard of before and they can make good stuff as well, and it's better to just try new things and see if they work for you, maybe. (Participant 17)

- cost of an intervention was perceived as an indicator of quality by some – “…cause it means that they have invested more time and money into it” (Participant 24). Others did not share this belief – “they're just trying to rip you off”

(Participant 5).

Students were questioned about the influence of people (family members, friends or famous people), or organizational endorsement of health claims (such as medical or government authorities). Opinions were mixed about the endorsement by a family member or a close friend. Some felt this indicated believability:

…if a friend says that it works, then I'd more believe them because I know them and I know where they come from. (Participant 3)

Others were less trusting of such endorsements, suggesting that even if an intervention works well for one person, it may not for another:

… every person is different and it might work on someone else, but if it were tried on another person, their body is different so it won't work exactly the same. (Participant 18)

Students reported that they generally believed claims that were endorsed by “unknown” consumers or “ordinary people” – “… cause it's straight from their experiences with it, not scripting and getting told what to say” (Participant 20).

For some, a celebrity endorsement could make a claim more believable:

Well, you kind of believe it a bit more because, like, the person has high standards because they're obviously a celebrity, so they're rich and everything, so I'd probably believe it a little bit more, not as much as, like, it has to be like, completely correct, but more than just a normal person. (Participant 6)

Yet, others were suspicious of celebrity endorsements, and expressed awareness about financial incentives:

… they're probably just saying it just to get the money, and it doesn't really feel like they're actually meaning it. (Participant 18)

While in some cases, celebrity endorsement made no difference to whether students believed the claim, if it came from a celebrity *health professional*, some students were sceptical – “wouldn't fully believe everything” (Participant 18). However, the endorsement of health information by any health professional (not necessarily celebrities) was generally viewed positively by students:

[I'd believe it] if a doctor had said it or, yeah, probably a doctor or someone qualified enough to prove that it is ‘true’. (Participant 21)

#### 3.1.3 Theme 3: Uncertainty about, and literal interpretation of, the language of health claims

Students associated terminology such as “‘evidence‐based,”’ “clinically proven” and “scientifically tested” with the assumption that research had been performed, or there was evidence or proof to support the claim:

So it means that the research has been done, so there's evidence that… the product is good or bad for you. (Participant 15)

Most students interpreted these terms literally – “[Evidence‐based means]… they've got evidence and it's based on what people have said, I think, yeah” (Participant 5).

A few offered a more detailed description:

… it [‘evidence‐based’] might be like they'll take the findings, they'll get like a bunch of test participants to sort of, like, test it and see if it works and sort of … or they'll give out, like, some people to do a trial of it for, like, 30 days and if they notice a difference or whatever, then they'll probably be like, yeah, ‘it works’. (Participant 14)

I guess it [‘evidence‐based’] means that they have tested it … so they haven't just tested it once or twice, they've tested it multiple times and took sort of everything into consideration or as much as they could, so a couple variables they've done. So, people with certain allergies, people without any, and yeah. (Participant 12)

However, most students acknowledged they did not understand the meaning of such terms:

Clinically proven. It's, sort of, like – I don't know about this one. Yeah. I'm not quite sure about this. (Participant 20)

Despite not fully understanding the terms, many viewed the intervention positively, stating that they would be more likely to use it:

I don't know what ‘clinically’ means, but I see [clinically proven] on everything and I'm just, like, oh, yeah, that'll be fine to use. (Participant 5)

It, sort of does to me [makes me more likely to use it], ‘cause it's one of those things that I feel ties in with a bit of the science and that behind it so it's been proven definitely. (Participant 20)

When asked how they thought new interventions compared to existing interventions, about half of the students responded that newer ones were better, with some elaborating that “newer” meant that more research, or “testing,” would have enhanced the newer one:

…there's more studies and research done and they've improved it probably. (Participant 12)

Others expressed uncertainty about whether new interventions were generally better, and a few students perceived new interventions negatively, explaining:

…some new treatments work as well, but just not as reliable as the old ones that have been used for a long time. (Participant 21)

Sometimes sticking with the old thing … is sometimes more reliable ‘cause … it's tested over years, but sometimes new ones might not be correct until a few years of testing… (Participant 20)

When asked whether, in general, new interventions have more or fewer side‐effects than older ones, responses were mixed. About half were unsure; some indicated that new interventions have more side‐effects – “… because they're newer and they don't have as much experience with the things that they're putting in there…” (Participant 1); while others believed that new interventions have less – “… [with] a lot of the new treatments, there's more side effects to start with but they sort of work out all the bugs and sort of get it good, whereas older ones generally had a lot more side effects…” (Participant 14).

## 4 DISCUSSION

We found that this sample of Australian school students, aged between 12‐15 years, generally had poor understanding about how health information and claims are generated and disseminated, and subsequently, how they can be assessed. Not unsurprisingly, many were largely reliant on their parents to manage any health conditions and students typically had little interaction with the health sector. However, many had already been exposed to health information and claims, and decision‐making about interventions which claim to impact upon health (for example, skin care products and sports drinks).

Many were generally sceptical about health information and claims, typically proffering concerns about conflict of interests, particularly financial, and the unregulated nature of the Internet, which allows anything to be published. Despite using terms such as “evidence” and “studies” in some of their replies, participants could not elaborate on what these terms mean or how to judge the accuracy of health claims. Instead they relied on personal experience or substitute indicators of accuracy, such as endorsements. Endorsements by friends, family or celebrities, appeared to be less consistently valued than those by health professionals and members of the public. Other substitute indicators included reading information on the product itself or associated websites, where descriptions of how the intervention works lent credibility, as did the quality of information presentation, the familiarity of the brand and the cost. An association of trust with familiar branding was noted in a study that examined health literacy challenges facing adolescents.^30^ The variety of responses illustrates the diversity of approaches used and assumptions made by students.

The use of research terms, such as “evidence‐based” and “clinically proven”, in health claims has become common. While many had previously heard or seen terms like these, students’ understanding of what the terms meant was superficial. There was dissonance between students’ acknowledged lack of understanding of the meaning, yet an inclination to trust interventions that used the terms. This phenomenon of the mere presence of a health claim (regardless of its accuracy or a potential users’ understanding of it) encouraging a positive perception of the intervention, has been previously noted in studies assessing food products and cigarettes.^42^, ^43^, ^44^, ^45^

We are not aware of any studies that have explored school students’ general understanding of, and approaches to, assessing health information and claims. However, some of the findings of this current study are similar to those found in studies of adults’ health information‐seeking behaviour. Studies of adults have found that while people have easy access to health information through the Internet and may perceive their “research” skills as good, they actually have difficulty judging the trustworthiness of health information.^31^, ^33^, ^46^, ^47^, ^48^ The use of the Internet to assess health information and claims in an unstructured way has also been previously found, with people often relying on search engines to identify relevant information,^12^, ^31^, ^33^ making personal judgements about the quality of the information using factors such as the information source and presentation,^12^, ^13^ and not considering the evidence about intervention effectiveness when making a decision.^49^ Adults searching for medicine information have also been found to search for corroborating information to reinforce a particular belief.^33^

Recent reviews of interventions to improve school students’ ability to assess health claims have found limited interventions in this area,^50^, ^51^ leaving students likely to inadvertently rely on inaccurate information when making health decisions.^18^, ^37^ This study has identified specific areas requiring attention, and the findings will assist us in developing and evaluating a school educational intervention, which aims to enable students to critically evaluate information about health interventions and claims.

Some of our findings are encouraging for the potential of using education to focus on the areas in which students have low understanding and skills. For example, many students were generally sceptical about health claims, with an awareness of ulterior motives and vested interests. They were already readily using the Internet to search for information and most had an awareness of the unregulated nature of the Internet – this may serve as an incentive to develop better skills in searching for and assessing the accuracy of health information and claims. Likewise, some students based their judgement of health claims either on personal experience or on triangulated information (similar information from multiple sources which students took as reinforcement of its validity). Teaching could expand upon these assumptions (that is, of multiple sources of information vs a single experience) to include the basics of research study hierarchies, what systematic reviews and randomized trials are, and why they are more believable than anecdotes from one person when assessing health claims. Students are unlikely to otherwise learn about key concepts^14^ such as these and having this knowledge has the potential to immediately influence their searching and interpretation behaviour.

Limitations of this study include possible unrepresentativeness of the sample. Boys and students from rural areas were under‐represented, and recruitment may have overrepresented middle‐ to high‐ socioeconomic status urban schools and students. Responses from participants may not accurately reflect actual behaviour, and we were unable to validate claims about their behaviours.

## 5 CONCLUSION

This study has provided insight into students’ understanding of issues relevant to assessing the accuracy of health information and claims and highlighted areas to incorporate into educational interventions. This sample of school students lacked understanding of basic health research processes and the knowledge or skills to assess health claims. This topic has had almost no attention in traditional school curricula, despite an increasing focus on teaching critical thinking in school subjects.^52^, ^53^, ^54^, ^55^, ^56^ There is growing recognition of the role of such skills in equipping adults with critical health literacy,^10^, ^11^, ^57^ and the school system may be an ideal place to begin teaching these skills. Until students (and adults) have this knowledge and skill set, they remain vulnerable to inaccurate and misleading health information and claims, which may result in them making ill‐informed health decisions.

## CONFLICT OF INTEREST

The authors declare that they have no conflict of interests relevant to this manuscript.
